# Eye-tracking-aided digital system for strabismus diagnosis

**DOI:** 10.1049/htl.2016.0081

**Published:** 2018-01-26

**Authors:** Zeng Hai Chen, Hong Fu, Wai Lun Lo, Zheru Chi, Bingang Xu

**Affiliations:** 1 Department of Computer Science, Chu Hai College of Higher Education, Tsuen Wan, Hong Kong; 2 Department of Electronic and Information Engineering, The Hong Kong Polytechnic University, Hung Hom, Kowloon, Hong Kong; 3 Faculty of Applied Science and Textiles, The Hong Kong Polytechnic University, Hung Hom, Kowloon, Hong Kong

**Keywords:** vision defects, patient diagnosis, gaze tracking, eye-tracking-aided digital system, strabismus diagnosis, vision disorders, preschool children, amblyopia, permanent vision loss, eye gaze

## Abstract

Strabismus is one of the most common vision disorders in preschool children. It can cause amblyopia and even permanent vision loss. In addition to a vision problem, strabismus brings to both children and adults serious negative impacts in their daily life, education, employment etc. Timely diagnosis of strabismus is thus crucial. However, traditional diagnosis methods conducted by ophthalmologists rely significantly on their experiences, making the diagnosis results subjective. It is also inconvenient for those methods being used for strabismus examination in large communities such as schools. In light of that, in this Letter, the authors develop an objective, digital and automatic system based on eye-tracking technique for diagnosing strabismus. The system exploits eye-tracking technique to acquire a person's eye gaze data while he or she is looking at some targets. A group of features are proposed to characterise the gaze data. The person's strabismus condition can be diagnosed according to the features. A strabismus gaze dataset is built using the system. Experimental results on the dataset demonstrate the effectiveness of the proposed system for strabismus diagnosis.

## 1 Introduction

Strabismus, termed squint and heterotropia as well, is that the two eyes do not point to the same direction. It is a common ophthalmic disorder with a prevalence of around 4% of the adult population [1]. If strabismus is not well treated, it would result in amblyopia or weak three-dimensional (3D) perception [2]. In addition to a vision problem, strabismus has been shown to have adverse psychosocial consequences in both children and adults [3]. For many young patients, their strabismus problems could be well treated or significantly alleviated if diagnosis and treatment are taken early. Therefore, timely diagnosis of strabismus is essential.

Traditional strabismus diagnosis methods include cover test, Hirschberg test, Maddox rod etc. These methods are conducted manually by the ophthalmologists according to their experiences. The diagnosis correctness is remarkably influenced by the ophthalmologist's professional qualities. Furthermore, these methods are manual, which makes it inconvenient to use for large communities (e.g. schools). In view of that, we propose and develop a digital strabismus diagnosis system based on eye-tracking technique in this Letter. The system is non-invasive and objective, and the diagnosis is generated automatically without any ophthalmologist's instruction. This makes it easy to carry out strabismus examination in large communities. For example, we can place the system in a primary school and the students can take their examinations at any time.

In the proposed system, we first show the subject nine target points ordered in different positions on a screen, and record the subject's gaze data. Then, a group of features are proposed to characterise the gaze data. A diagnosis result is finally made by analysing the features. We build a gaze dataset using our eye-tracking system for performance evaluation. The effectiveness of our method is demonstrated by comparing the diagnosis result with professional ophthalmologist's diagnosis result.

The rest of this Letter is organised as follows. We first review Letters on the eye-tracking methodology for solving various problems, especially disease detection and analysis, and then describe the proposed system and elaborate the features that are used to characterise gaze data. After that, we introduce our dataset and report experimental results. A conclusion is drawn at the end of this Letter.

## 2 Related work

Approaches have been developed to detect eye movements since the last century. For example, Jolson *et al.* [4] proposed an apparatus for evaluating the alignment of both eyes. Knapp *et al.* [5] developed a method and apparatus for determining the position of each eye of a human with respect to a fixed reference point or with respect to each other by using electro-oculogram signals produced by eye movement, so as to measure strabismus. Compared to modern eye trackers, however, the accuracy of this apparatus is pretty low. In [6], Schaeffel developed an automated procedure to measure kappa and the Hirschberg ratio for immediate use in a video gaze tracker. Yang *et al.* [7] used a 3D strabismus photo analyser to estimate binocular alignment using photographs, and found that the 3D Strabismus Photo Analyser is a simple and reliable tool for measuring ocular deviation.

Thanks to its rapid development, the eye-tracking technique has been successfully applied to solving various problems, e.g. object recognition [8], attention modelling [9], image quality assessment [10], and biometric identification [11], as well as disease investigation [12, 13].

There are some attempts that leverage eye-tracking methodology for strabismus examination [14–18]. In the last century, people have attempted to build automatic eye-tracking systems for measuring strabismic deviation based on television cameras [14, 15]. In the past several years, people continued to refine strabismus examination by developing more advanced eye-tracking methods. For example, Pulido [16] used Tobii eye tracker to acquire gaze data, and calculated the deviation of gaze data for ophthalmic diagnostics including strabismus. However, Pulido proposed a method prototype only. The author had no real strabismic gaze data to demonstrate the prototype's performance. Model and Eizenman [17] proposed an automatic method based on eye-tracking for performing the Hirschberg test, a classical method to measure binocular ocular misalignment. To precisely locate the pupil centres and corneal reflexes, they built a remote binocular gaze-tracking system using two video cameras and three infrared light sources. However, the performance was studied with five healthy infants only. Thus, the method's effectiveness for strabismus examination had not been tested. In Model's doctoral dissertation, he proposed two personal calibration procedures that do not require active user participation for estimation of gaze point and measurement of ocular alignment for adults and infants [18], but his methods were not been tested with sufficient strabismic data. Bakker *et al.* [19] developed a gaze direction measurement instrument to estimate strabismus angle. The instrument allows for unrestrained head movement by means of a triple camera vision system that simultaneously estimates the head rotation and the eye pose. Only three subjects participated in the experiment.

In recent year, virtual reality technique is also applied to eye health. For instance, Blaha and Gupta [20] designed a virtual reality game to help people with amblyopia restore vision, but they focused on amblyopia training rather than diagnosis.

## 3 Methodology

**Fig. 1:**
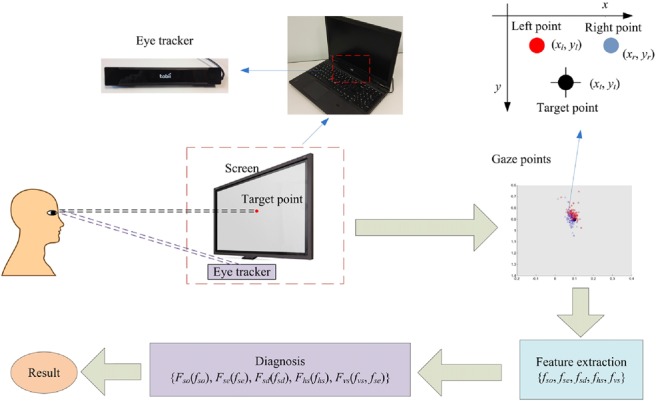
Framework of the proposed strabismus diagnosis system

Fig. 1 shows the framework of the proposed system, in which the diagnosing procedure for a subject is carried out as follows:

- The subject sits at a fixed near distance from a laptop with an eye tracker mounted below the laptop's screen.
- The subject fixates on a number of calibration points to perform calibration.
- The subject fixates on a number of target points for eye gaze data collection.
- A group of features are extracted from all the gaze data.
- A diagnosis result is made according to the features.

The above procedures will be detailed in the subsequent sections.

### 3.1 Gaze data acquisition

#### 3.1.1 Eye-tracking system

**Fig. 2:**
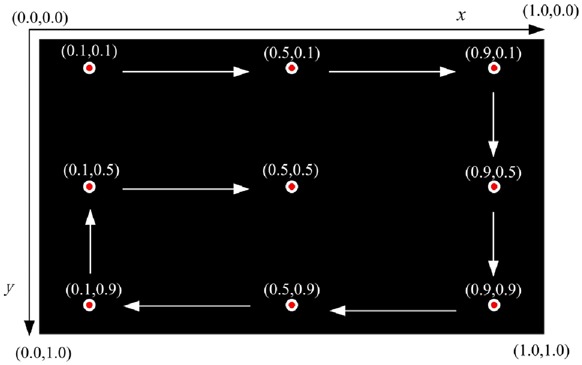
Nine-point gaze data acquisition interface. The white arrows indicate the display order

We use eye tracker Tobii X2-60 to build our system. Its sampling rate is 60 Hz and tracking accuracy is 0.4°. To make the system portable, we mount the eye tracker below the monitor of a laptop, as shown in Fig. 1. The laptop is Lenovo Thinkpad T540p with a 1920 × 1080 screen resolution. We employ Tobii MATLAB SDK to design our experiments for gaze data acquisition. The coordinate system of the screen is defined as follows. The upper-left corner of the screen is set as the origin, the coordinate value of which is (0, 0), with horizontal denoting *x*-coordinate and vertical denoting *y*-coordinate. The coordinate value of the lower-right corner is (1, 1), and the values of the upper-right corner and lower-left corner are (1, 0) and (0, 1), respectively. Both the *x-*value and *y-*value of any position on the screen are between 0 and 1, as shown in Fig. 2.

#### 3.1.2 Calibration

The purpose of calibration is to teach the eye-tracking system the characteristics of the subject, such that the eye tracker can well detect the subject's eye movements in a subsequent test. In the calibration process, we adjust the subject's chair and the eye-tracking screen to make sure that the subject is at a fixed distance (50 cm in our experiments) from the screen and his or her eye level is horizontally pointing to the screen centre, i.e. point (0.5, 0.5). We choose a distance of 50 cm as it is an optimal distance for eye tracker Tobii X2-60 to track the subject's eye movements. We use nine-point calibration scheme. If the calibration result indicates that the fixation accuracy of either eye is acceptable (average accuracy higher than a predefined value, e.g. 0.05 in this Letter), we can then start the subsequent test. Otherwise, we should recalibrate.

#### 3.1.3 Data acquisition

A classical way for the ophthalmologist to examine strabismus is a nine-point method. That is, the ophthalmologist instructs the patient to fixate on nine points at a certain distance in front, and then observes the patient's eye movements. The nine-point method can comprehensively examine one's eye movements with rotations at different angles. We adopt the same method to design our gaze data acquisition interface. The coordinate values of the nine points that we used are the same for the nine calibration points. The points are displayed one by one orderly. Fig. 2 shows the nine-point gaze data acquisition interface. The white arrows point out the display order. A black background of the interface helps the subject to concentrate on the target points. Data acquisition programme runs as follows. Each time one target point is displayed. Meanwhile, the subject's gaze points are recorded by eye tracker. If the number of effective gaze pairs acquired exceeds 100, current target point is removed and the next target point would be displayed. A gaze pair is defined as two gaze points of the two eyes captured by eye tracker at one sampling moment. ‘Effective’ here means that at least one gaze point of a gaze pair locates close enough to the target point. That is, the distance between the target point and either gaze point of a gaze pair must be smaller than a threshold (0.05 in this Letter) predefined empirically. For those who suffer serious strabismus, it is sometimes hard to capture effective gaze points at some target points, e.g. points at the four corners of the screen, because these points need strabismic subjects to rotate eyeballs to their extreme. To handle this case, we let eye tracker record gaze points at each point for at most 10 s. The next target point would be displayed after 10 s no matter whether or not the system has collected 100 pairs of gaze points. The sampling rate of our eye tracker is 60 Hz. It takes only 2 s to collect 100 gaze pairs for normal people. Thus, 10 s are long enough to capture gaze data for each point.

### 3.2 Feature extraction for diagnosis

The next step after gaze data acquisition is feature extraction from all the gaze points. On the basis of the features, we should be able to tell whether the subject has strabismus or not, which eye is strabismic, and what type of strabismus it is. The features for answering these questions are constructed and described as follows.

#### 3.2.1 Strabismus occurrence feature

**Fig. 3:**
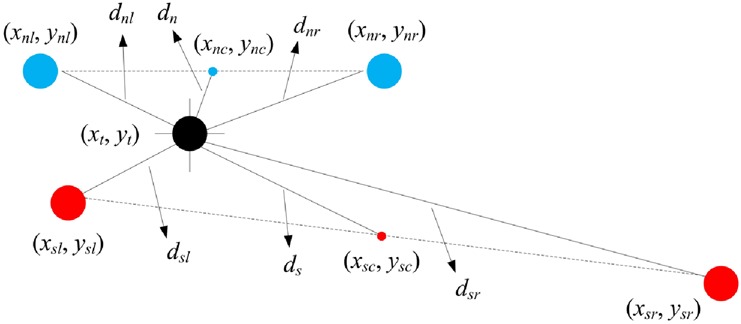
Example of normal (blue) and strabismic (red) gaze pairs, and their target point (black)

Strabismus occurrence feature is defined to answer the question of whether or not the subject has strabismus. To realise that we propose to exploit two features: fixation deviation and fixation contrast. Fixation deviation is defined as the Euclidean distance between the centre of a gaze pair and the target point. We can also calculate the Euclidean distance between left or right gaze point and the target point. Fixation contrast is then defined as the difference between the Euclidean distances of left gaze point and right gaze point to the target point. Fig. 3 shows an example of a normal gaze point pair (blue) and a strabismic gaze point pair (red) together with their target point (black). (*x*_nl_, *y*_nl_), (*x*_nr_, *y*_nr_), and (*x*_nc_, *y*_nc_) denote left gaze point, right gaze point, and the centre of a normal gaze pair, and (*x*_sl_, *y*_sl_), (*x*_sr_, *y*_sr_), and (*x*_sc_, *y*_sc_) are corresponding points for a strabismic gaze pair. (*x*_t_, *y*_t_) is the target point. *d*_nl_, *d*_nr_, *d*_n_, *d*_sl_, *d*_sr_, and *d*_s_ are distances between the target point and corresponding points. The significant difference between a normal gaze pair and a strabismic gaze pair is that the strabismic pair has always a point close to the target point and the other one relatively far from the target point, while the two points of the normal pair locate at a similar distance from the target point. As a result, the strabismic pair usually has a fixation deviation *d*_s_ larger than that (*d*_n_) of the normal pair, and a fixation contrast |*d*_sl_−*d*_sr_| larger than that (|*d*_nl_−*d*_nr_|) of the normal pair. Therefore, we can use fixation deviation and fixation contrast to classify strabismic gaze data and normal gaze data accordingly.

Mathematically, let (*x*_l_, *y*_l_) and (*x*_r_, *y*_r_) denote the left gaze point and right gaze point for a gaze pair, and (*x*_t_, *y*_t_) denote the target point. We can then have the formulation of fixation deviation defined as below:

(1) $$f_{\rm d} = \sqrt {{\left({\displaystyle{{\left({x_{\rm l} + x_{\rm r}} \right)} \over 2} - x_{\rm t}} \right)}^2 + {\left({\displaystyle{{\left({y_{\rm l} + y_{\rm r}} \right)} \over 2} - y_{\rm t}} \right)}^2} \comma \; \eqno\lpar 1\rpar $$

and the formulation of fixation contrast defined as below:

(2) $$f_{\rm c} = \left\vert {d_{\rm l} - d_{\rm r}} \right\vert \comma \; \eqno\lpar 2\rpar $$

where

(3) $$d_{\rm l} = \sqrt {{\left({x_{\rm l} - x_{\rm t}} \right)}^2 + {\left({y_{\rm l} - y_{\rm t}} \right)}^2} \eqno\lpar 3\rpar $$

and

(4) $$d_{\rm r} = \sqrt {{\left({x_{\rm r} - x_{\rm t}} \right)}^2 + {\left({y_{\rm r} - y_{\rm t}} \right)}^2} \eqno\lpar 4\rpar $$

We calculate *f*_d_ and *f*_c_ for all the gaze pairs of nine target points. A simple way to obtain a representative value for all the gaze data is to average *f*_d_ and *f*_c_ over all the gaze pairs. However, the average result would be dominated by some extreme gaze points, which cannot represent well the subject's normal fixation state. To alleviate the effect of extreme points, we take into account a statistical measure of percentile for the calculations of *f*_d_ and *f*_c_. A percentile indicates the value below which a given percentage of observations fall. For example, the 60th percentile of *f*_d_ is the value below which 60% of gaze pairs’ *f*_d_ may be found. It can characterise the major gaze pairs’ *f*_d_. We tested percentiles for both *f*_d_ and *f*_c_ from 70 to 95 and found that the diagnosis results do not vary much. In this Letter, for a convenient comparison, we present the widely used 85th percentile only. We denote the percentiles of *f*_d_ and *f*_c_ over all the gaze data of nine target points as *f*_dp_ and *f*_cp_.

For some strabismic people, their eyes can fixate precisely at some directions, whereas for some other directions the two eyes do not align well. This case happens a lot in particular to incomitant strabismus, the strabismic eye of which cannot move in some specific directions. That is why the ophthalmologist needs to use the nine-point method to examine the patient's eye movements. Taking this case into account, we add two features called maximum fixation deviation *f*_dm_ and maximum fixation contrast *f*_cm_. We first calculate 85th percentile of *f*_d_ and *f*_c_ for each target point. Then, max fixation deviation *f*_dm_ is defined as the maximum value of 85th percentile of *f*_d_ over the nine target points, and max fixation contrast *f*_cm_ is defined as the maximum value of 85th percentile of *f*_c_ over the nine target points. *f*_dm_ and *f*_cm_ can be formulated as follows:

(5) $$f_{{\rm dm}} = \max \lpar f_{{\rm dp}}^{\lpar i\rpar } \rpar \quad i \in \lpar 1\comma \; \, \ldots \comma \; \, 9\rpar \eqno\lpar 5\rpar $$

and

(6) $$f_{{\rm cm}} = \max \lpar f_{{\rm cp}}^{\lpar i\rpar } \rpar \quad i \in \lpar 1\comma \; \, \ldots \comma \; \, 9\rpar \eqno\lpar 6\rpar $$

where $f_{{\rm dp}}^{\lpar i\rpar } $ and $f_{{\rm cp}}^{\lpar i\rpar } $ represent, respectively, the 85th percentiles of *f*_d_ and *f*_c_ for the *i*th target point.

We can have strabismus occurrence feature *f*_so_ by combining features *f*_dp_, *f*_cp_, *f*_dm_, and *f*_cm_ as follows:

(7) $$f_{{\rm so}} = k_1f_{{\rm dp}} + k_2f_{{\rm cp}} + k_3f_{{\rm dm}} + k_4f_{{\rm cm}} - \alpha \comma \; \eqno\lpar 7\rpar $$

where *k*_1_, *k*_2_, *k*_3_, and *k*_4_ are coefficients used to weigh the four features. They can be determined according to practical requirements. In this Letter, *k*_1_ = *k*_2_ = *k*_3_ = *k*_4_ = 1. Constant *α* is used to bias the output, such that *f*_so_ > 0 represents strabismus and *f*_so_ < 0 represents normal. Constant *α* may be calculated by different algorithms. The algorithm we adopt is to compute *f*_dp_ + *f*_cp_ + *f*_dm_ + *f*_cm_ for both normal subjects and strabismic subjects. Moreover, then the largest value of normal subjects and the smallest value of strabismic subjects are selected. Their average value is assigned to *α*. We calculate *α* based on the data attained. This mechanism is reasonable, as many medical and health indicators are decided by analysing a large number of healthy and unhealthy people's data. With strabismus occurrence feature *f*_so_ defined in (7), the decision function is finally defined as

(8) $$F_{{\rm so}}\lpar f_{{\rm so}}\rpar = \left\{{\matrix{ 1 & {\,f_{{\rm so}} \gt 0} \cr 0 & {\,f_{{\rm so}} \le 0} \cr } } \right.\eqno\lpar 8\rpar $$

where *F*_so_ = 1 indicates the subject has strabismus and *F*_so_ = 0 indicates the subject is normal.

#### 3.2.2 Strabismic eye feature

After the subject is diagnosed to have strabismus with strabismus occurrence feature, the next step is to work out which eye is strabismic. The feature used to solve the problem is called strabismic eye feature, denoted by *f*_se_. Its definition is as follows:

(9) $$f_{{\rm se}} = \displaystyle{1 \over N}\sum\limits_{i = 1}^N {\left({d_{\rm l}^{\lpar i\rpar } - d_{\rm r}^{\lpar i\rpar } } \right)} \comma \; \eqno\lpar 9\rpar $$

where $d_{\rm l}^{\lpar i\rpar } $ and $d_{\rm r}^{\lpar i\rpar } $ are the Euclidean distances between the target point and the left gaze point and right gaze point of the *i*th gaze pair, respectively. The formulas of $d_{\rm l}^{\lpar i\rpar } $ and $d_{\rm r}^{\lpar i\rpar } $ are the same in (3) and (4). *N* is the total number of gaze pairs acquired for the subject. The decision function of feature *f*_se_ is

(10) $$F_{{\rm se}}\lpar f_{{\rm se}}\rpar = \left\{{\matrix{ 1 & {\,f_{{\rm se}} \gt 0} \cr 0 & {\,f_{{\rm se}} \le 0} \cr } } \right.\eqno\lpar 10\rpar $$

Function *F*_se_ = 1 means that the right eye fixates better than the left eye, and thus the left eye is a strabismic eye, and *F*_se_ = 0 indicates the right eye is a strabismic eye.

#### 3.2.3 Strabismus direction feature

To determine which one of the four types (horizontal exotropia and esotropia and vertical hypertropia and hypotropia) the strabismus is, we should identify its direction (horizontal or vertical) first. The angle between the line connecting the two gaze points of a gaze pair and the horizontal line is utilised to estimate the strabismus direction. We call the angle strabismus direction feature, which is defined as follows:

(11) $$f_{{\rm sd}} = \arctan \left({\displaystyle{{\left\vert {y_{\rm l} - y_{\rm r}} \right\vert } \over {\left\vert {x_{\rm l} - x_{\rm r}} \right\vert }}} \right).\eqno\lpar 11\rpar $$

**Fig. 4:**
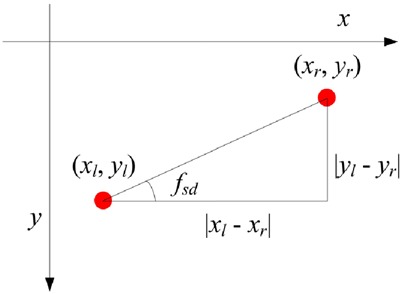
Strabismus direction feature f_sd_

Fig. 4 depicts the strabismus direction feature *f*_sd_ of a gaze pair. *f*_sd_ lies in the range [0°, 90°]. An intuitive method to determine the strabismus direction is to binarise *f*_sd_ with threshold 45°. *f*_sd_ > 45° signifies that the two eyes tend to fixate vertically, and it is vertical strabismus accordingly. Otherwise, it is assigned to horizontal strabismus. However, a slight (e.g. below 5°) vertical strabismus may not affect one's vision, and it is usually ignored in diagnosis. Therefore, most of the strabismus is diagnosed as exotropia or esotropia by ophthalmologists, even though some degrees of hypertropia or hypotropia may occur simultaneously. Only obvious vertical strabismus is assigned to hypertropia or hypotropia. Taking this situation into account, in our method, we evenly divide the range of *f*_sd_ into three intervals: [0°, 30°], [30°, 60°], and [60°, 90°], in which the strabismus is classified into horizontal, horizontal prior, and vertical. Horizontal prior means that the subject suffers both horizontal and vertical strabismus, but we still assign him or her to horizontal strabismus with priority in a binary decision. The decision function can be consequently formulated as

(12) $$F_{{\rm sd}}\lpar f_{{\rm sd}}\rpar = \left\{{\matrix{ 1 & {0^\circ \le f_{{\rm sd}} \le 30^\circ } \cr 0 & {30^\circ \le f_{{\rm sd}} \le 60^\circ } \cr { - 1} & {60^\circ \le f_{{\rm sd}} \le 90^\circ } \cr } } \right.\eqno\lpar 12\rpar $$

where *F*_sd_ = 1 denotes horizontal strabismus, *F*_sd_ = 0 denotes horizontal-prior strabismus, and *F*_sd_ = −1 denotes vertical strabismus. The decision result here is three-fold rather than two-fold. One big advantage of a three-fold decision is that it can provide more detailed information to ophthalmologists in treating strabismus, e.g. strabismus surgery. For example, if the decision result is *F*_sd_ = 0, the ophthalmologist knows that the patient has both horizontal and vertical strabismuses. In real diagnosis, *f*_sd_ will be also used as an important indicator of strabismus evaluation.

#### 3.2.4 Horizontal strabismus feature

The horizontal strabismus feature used to distinguish exotropia and esotropia is defined as

(13) $$f_{{\rm hs}} = \displaystyle{1 \over N}\sum\limits_{i = 1}^N {\left({x_{\rm r}^{\lpar i\rpar } - x_{\rm l}^{\lpar i\rpar } } \right)} - \beta \comma \; \eqno\lpar 13\rpar $$

where $x_{\rm l}^{\lpar i\rpar } $ and $x_{\rm r}^{\lpar i\rpar } $ are the *x* values of the left and right gaze points of the *i*th gaze pair. *N* is the total number of gaze pairs. Bias *β* is calculated by averaging ($x_{\rm r}^{\lpar i\rpar } - x_{\rm l}^{\lpar i\rpar } $) over all the normal subjects. The decision function is

(14) $$F_{{\rm hs}}\lpar f_{{\rm hs}}\rpar = \left\{{\matrix{ 1 & {\,f_{{\rm hs}} \gt 0} \cr 0 & {\,f_{{\rm hs}} \le 0} \cr } } \right.\eqno\lpar 14\rpar $$

where *F*_hs_ = 1 is exotropia and *F*_hs_ = 0 is esotropia. The magnitude of *f*_hs_ is a measurement of severity. A smaller magnitude of *f*_hs_ indicates a smaller severity of strabismus.

#### 3.2.5 Vertical strabismus feature

The vertical strabismus feature used to classify hypertropia and hypotropia is defined as

(15) $$f_{{\rm vs}} = \displaystyle{1 \over N}\sum\limits_{i = 1}^N {\left({y_{\rm r}^{\lpar i\rpar } - y_{\rm l}^{\lpar i\rpar } } \right)} \comma \; \eqno\lpar 15\rpar $$

where $y_{\rm l}^{\lpar i\rpar } $ and $y_{\rm r}^{\lpar i\rpar } $ are the *y* values of the left and right gaze points of the *i*th gaze pair. *N* is again the total number of gaze pairs. *f*_vs_ has a similar form with *f*_hs_, except for bias *β*. Bias *β* is used to eliminate the horizontal distance produced by pupil distance. *f*_vs_ tells the relative position of the two eyes horizontally. However, if we want to determine hypertropia or hypotropia, we need to find out which eye is a strabismic eye. In other words, we have to take into account both *f*_vs_ and *f*_se_ in order to make the decision. Mathematically, the decision function of vertical strabismus is defined as follows:

(16) $$F_{{\rm vs}}\lpar f_{{\rm vs}}\comma \; \, f_{{\rm se}}\rpar = \left\{{\matrix{ 1 & {\,f_{{\rm vs}} \times f_{{\rm se}} \gt 0} \cr 0 & {\,f_{{\rm vs}} \times f_{{\rm se}} \le 0} \cr } } \right.\eqno\lpar 16\rpar $$

where *F*_vs_ = 1 is hypertropia and *F*_vs_ = 0 is hypotropia. Similar to *f*_hs_, the magnitude of *f*_vs_ measures the severity of vertical strabismus, with a smaller value denoting a smaller severity.

#### 3.2.6 Diagnostic procedure

**Fig. 5:**
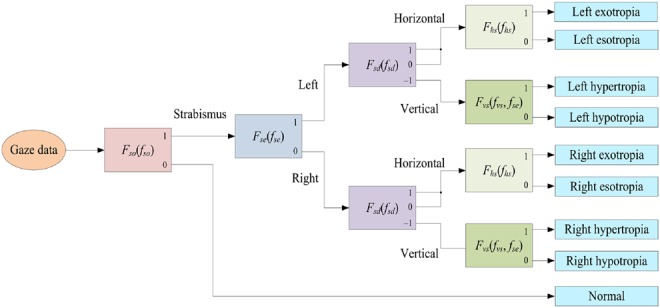
Diagnostic procedure using the proposed features

Fig. 5 depicts the whole diagnostic procedure using the proposed features extracted from the gaze data of a subject. First of all, strabismus occurrence feature *f*_so_ is used to estimate whether strabismus occurs or not. If strabismus occurs, strabismic eye feature *f*_se_ is exploited to figure out which eye is a strabismic eye. After that, strabismus direction feature *f*_sd_ is employed to determine the direction of strabismus, i.e. horizontal or vertical. If the result is horizontal, horizontal strabismus feature *f*_hs_ is used to work out the strabismus type, i.e. exotropia or esotropia. Otherwise, vertical strabismus feature *f*_vs_ is used to work out the vertical strabismus type, i.e. exotropia or esotropia. Our system can also provide relative severity of strabismus. The severity of strabismus can be measured by horizontal strabismus feature *f*_hs_ and vertical strabismus feature *f*_vs_. A larger value of these features indicates a more severe strabismus. In particular, *f*_hs_ and *f*_vs_ can be used to compare the severity of the same types of strabismuses.

## 4 Experiments

After ethics approval from Human Research Ethics Committee of Chu Hai College of Higher Education and informed consent, we collected gaze data from 15 normal subjects and 10 strabismic subjects. The age range of normal subjects is from 7 to 40, whereas the age range of strabismic subjects is from 3 to 63. The mean ages are 30 and 40 for normal subjects and strabismic subjects, respectively. Both male and female persons are involved. The numbers of females are 9 and 6 for normal subjects and strabismic subjects, respectively. We denote 15 normal subjects as *N*_1_–*N*_15_ and 10 strabismic subjects as *S*_1_–*S*_10_. The strabismic subjects have been diagnosed by professional ophthalmologists, and the results are used as ground truths. The ground truths of ten strabismic subjects are tabulated in Table 1. Table 1 reports strabismic eye, strabismus direction, type, and degree, such that we can have a clear comparison between the results generated by our system and the ground truths. The severity of strabismus for ten strabismic subjects can be roughly ranked as *S*_1_ > *S*_8_ > *S*_4_ > *S*_2_ > *S*_5_ = *S*_7_ > *S*_3_ = *S*_9_ > *S*_6_ = *S*_10_.

**Table 1:** Ground truths of ten strabismic subjects

Subject	Strabismic eye	Direction	Type	Degree
*S*_1_	left	vertical	hypertropia	18
*S*_2_	left	horizontal	esotropia	12
*S*_3_	right	horizontal	exotropia	6
*S*_4_	right	horizontal	exotropia	14
*S*_5_	right	horizontal	esotropia	8
*S*_6_	left	horizontal	esotropia	4
*S*_7_	right	horizontal	esotropia	8
*S*_8_	right	horizontal	esotropia	16
*S*_9_	left	vertical	hypertropia	6
*S*_10_	left	horizontal	exotropia	4

### 4.1 Experimental results

**Fig. 6:**
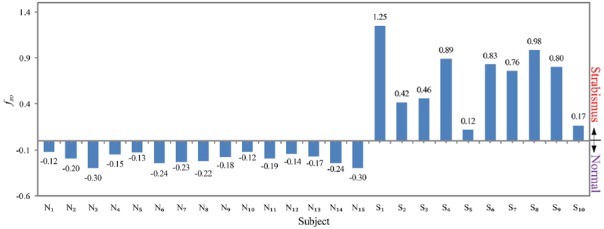
Strabismus occurrence feature f_so_ of 15 normal subjects and 10 strabismic subjects

Strabismus occurrence feature *f*_so_ is shown in Fig. 6. Bias *α* = 0.428. All the strabismic subjects have positive values, and all the normal subjects have negative values. Evidently, the strabismus occurrence feature *f*_so_ defined in (7) separates the normal data and strabismic data well. This indicates that feature *f*_so_ can effectively characterise the difference between gaze data of normal subjects and strabismic subjects. With a simple calculation, we can know that the mean values of *f*_so_ are −0.2 and 0.67 for normal subjects and strabismic subjects, and the standard deviations are 0.06 and 0.37 for normal subjects and strabismic subjects, respectively. This signifies that the intra-difference of normal subjects is much smaller than that of strabismic subjects, and the values of *f*_so_ are pretty diverse for strabismic subjects. To evaluate the difference between normal data and strabismic data statistically, we perform a *t*-test for the two groups of data. The *p*-value is 4.7 × 10^−9^, much smaller than 5% significant level, which means that the *f*_so_ values of normal subjects and strabismic subjects are significantly different. This indicates that feature *f*_so_ is an effective indicator to separate normal subjects and strabismic subjects.

**Fig. 7:**
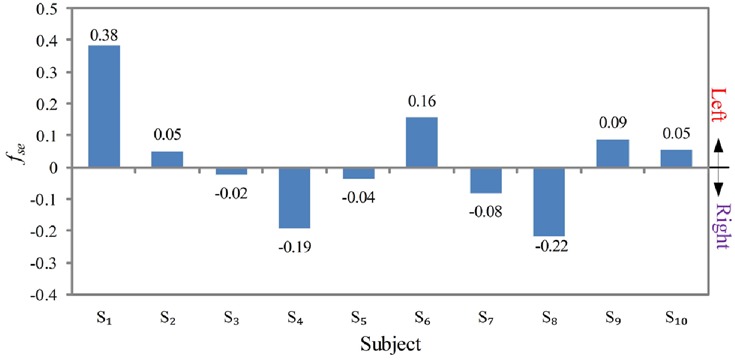
Strabismic eye feature f_se_ of ten strabismic subjects

**Fig. 8:**
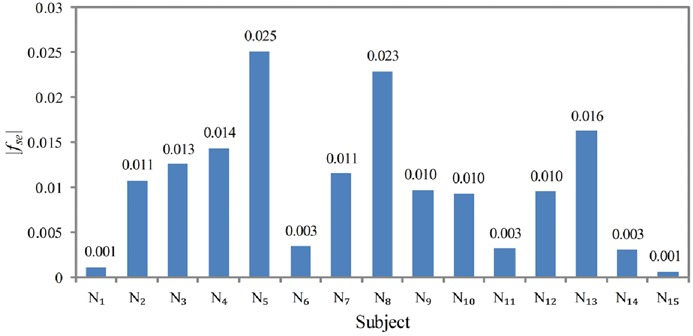
Magnitude |f_se_| of strabismic eye feature f_se_ of 15 normal subjects

After strabismus is confirmed, strabismic eye feature *f*_se_ is adopted to find out the strabismic eyes for ten strabismic subjects. Fig. 7 depicts the *f*_se_ values for ten strabismic subjects. Feature *f*_se_ > 0 indicates left eye is a strabismic eye. Therefore, we can see that for subjects *S*_1_, *S*_2_, *S*_6_, *S*_9_, and *S*_10_, their left eyes are strabismic eyes, and for subjects *S*_3_, *S*_4_, *S*_5_, *S*_7_, and *S*_8_, their right eyes are strabismic eyes. The results are consistent with the ground truths as shown in Table 1. We perform a *t*-test for the two groups (left strabismic eye and right strabismic eye). The *p*-value is 0.0088, demonstrating that the feature *f*_se_ of two groups are significantly different. Note that the magnitude of *f*_se_ signifies the fixation difference between the left eye and right eye. A smaller |*f*_se_| stands for more similar fixation accuracies that the two eyes have. To further investigate that we show |*f*_se_| for 15 normal subjects in Fig. 8. One important finding is that all the normal subjects have a small magnitude of *f*_se_. The largest one is not larger than 0.025. This is because those normal subjects possess good fixation capabilities for both eyes.

**Fig. 9:**
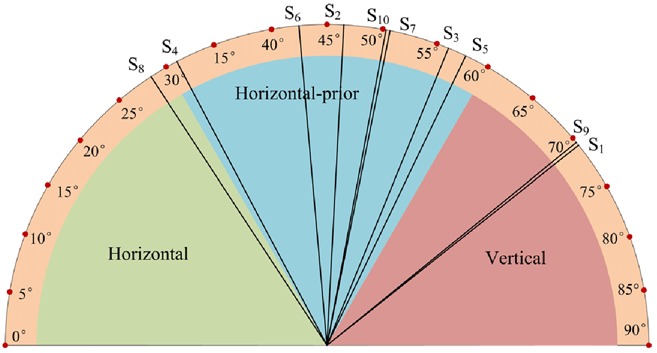
Strabismus direction feature f_sd_ of ten strabismic subjects

The next step is to find out strabismus direction, e.g. horizontal or vertical, using feature *f*_sd_. Fig. 9 depicts the values of *f*_sd_ for ten strabismic subjects. The three intervals of *f*_sd_ are differentiated by three colours in the sector. We can estimate that the strabismus directions of *S*_1_ and *S*_9_ are vertical, *S*_8_ is horizontal, *S*_2_, *S*_3_, *S*_4_, *S*_5_, *S*_6_, *S*_7_, and *S*_10_ are horizontal prior, which could be finally classified to horizontal. In real diagnosis, the value of *f*_sd_ will be outputted to the doctors for reference. The *p*-value of *t*-test for the vertical and horizontal (including horizontal prior) data is 0.008, which demonstrates the significant difference between vertical and horizontal data, and the effectiveness of feature *f*_sd_ in representing strabismus direction.

**Fig. 10:**
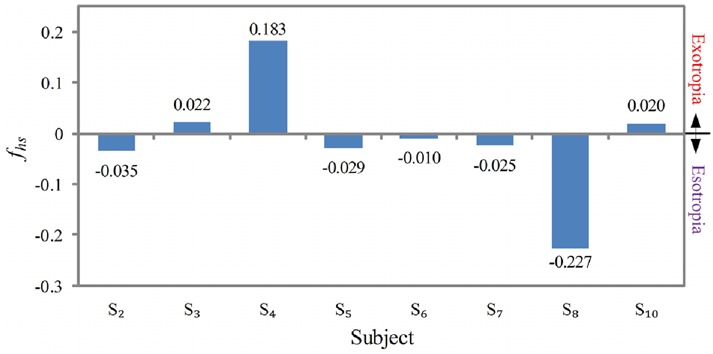
Horizontal strabismus feature f_hs_ of eight subjects that have horizontal strabismuses

We can then use horizontal strabismus feature *f*_hs_ or vertical strabismus feature *f*_vs_ to work out strabismus type. Let us investigate *S*_2_, *S*_3_, *S*_4_, *S*_5_, *S*_6_, *S*_7_, *S*_8_, and *S*_10_ that have been classified to horizontal strabismus first. Fig. 10 shows their *f*_hs_. Obviously, *S*_3_, *S*_4_, and *S*_10_ are exotropia, and *S*_2_, *S*_5_, *S*_6_, *S*_7_, and *S*_8_ are esotropia. The results are consistent with Table 1. Their *p*-value is 0.08, larger than 5% significant level. In other words, the difference between *f*_hs_ value of exotropia and that of esotropia is not significant enough. The magnitude of *f*_hs_ measures strabismus severity. A larger magnitude of *f*_hs_ represents a more serious strabismus. The subjects’ strabismus severity can be thus ranked as *S*_8_ > *S*_4_ > *S*_2_ > *S*_5_ > *S*_7_ > *S*_3_ > *S*_10_ > *S*_6_. As shown in Table 1, the ground truth order is *S*_8_ > *S*_4_ > *S*_2_ > *S*_5_ = *S*_7_ > *S*_3_ > *S*_10_ = *S*_6_. The difference between the order generated by our system and the ground truth is that *S*_5_ and *S*_7_ and *S*_10_ and *S*_6_ are diagnosed by ophthalmologists to have the same degrees, while our system diagnoses that *S*_5_ is more severe than *S*_7_ and *S*_10_ is severe than *S*_6_.

**Fig. 11:**
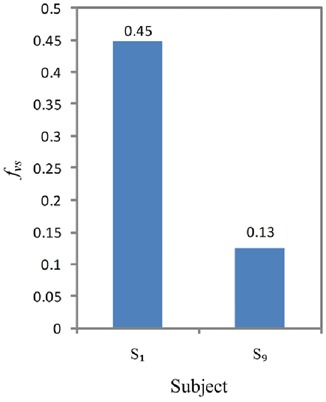
Vertical strabismus feature f_vs_ of two subjects that have vertical strabismuses

Fig. 11 shows vertical strabismus feature *f*_vs_ of *S*_1_ and *S*_9_. Note that vertical strabismus type is determined by the product of *f*_se_ and *f*_vs_ in (16). It can be calculated from Figs. 7 and 11 that *f*_se_ × *f*_vs_ > 0 for both *S*_1_ and *S*_9_. According to (16), *S*_1_ and *S*_9_ are hypertropias. Moreover, the magnitude of *f*_vs_ signifies that the severity ranking is *S*_1_ > *S*_9_. This is consistent with the ground truth.

Overall, the experimental results demonstrate that our method is able to diagnose out all the normal and strabismic subjects using eye-tracking data. The results are consistent with ophthalmologist's diagnosis reports.

## 5 Conclusion

In this Letter, we propose and develop an intelligent digital system based on eye-tracking technique for objective and automatic strabismus diagnosis. An eye-tracking system is first developed to acquire people's gaze data. A group of features are then proposed to characterise the gaze data. On the basis of these features, the proposed system is able to tell whether or not the subject has strabismus, which eye is strabismic, what type of strabismus it is, and the severity of strabismus. We build a gaze dataset that contains both normal and strabismic gaze data for experiments. The results generated by our system are consistent with those diagnosed by ophthalmologists. All experimental results have demonstrated the effectiveness of our method. More importantly, the proposed system is objective, non-invasive, and automatic. It could be a potential powerful alternative for strabismus diagnosis.

Compared to other automatic strabismus diagnosis systems, one major advantage of our system is its portability and convenience. We can easily bring the system to different places to conduct strabismus examination for people. The setup is very simple; meanwhile, high accuracy can be still achieved for diagnosing strabismus occurrence and types. The limitation of our system is that it cannot yet precisely measure the strabismus angle as the cover test with a prism, though the relative severity is quite accurate. In the future work, we will develop more effective features for precise strabismus degree evaluation.

## References

[1] Coats D.K. Stager D.R. Sr. Beauchamp G.R.: ‘Reasons for delay of surgical intervention in adult strabismus’, Arch. Ophthalmol., 2005, 123, pp. 497–499 (doi: 10.1001/archopht.123.4.497) 15824223 10.1001/archopht.123.4.497

[2] Mojon-Azzi S.M. Kunz A. Mojon D.S.: ‘The perception of strabismus by children and adults’, Graefes Arch. Clin. Exp. Ophthalmol., 2011, 249, (5), pp. 753–757 (doi: 10.1007/s00417-010-1555-y) 21063886 10.1007/s00417-010-1555-y

[3] Durnian J.M. Noonan C.P. Marsh I.B.: ‘The psychosocial effects of adult strabismus: a review’, Br. J. Ophthalmol., 2011, 95, (4), pp. 450–453 (doi: 10.1136/bjo.2010.188425) 20852320 10.1136/bjo.2010.188425

[4] Jolson A.S. Myler H.R. Weeks A.: ‘Apparatus for evaluating eye alignment’. U.S. Patent No. 5094521, 1992

[5] Knapp R.B. Hake L.E. Lusted H.S.: ‘Method and apparatus for eye tracking for convergence and strabismus measurement’. U.S. Patent 5491492, 1996

[6] Schaeffel F.: ‘Kappa and Hirschberg ratio measured with an automated video gaze tracker’, Optom. Vis. Sci., 2002, 79, (5), pp. 329–334 (doi: 10.1097/00006324-200205000-00013) 12035991 10.1097/00006324-200205000-00013

[7] Yang H.K. Han S.B. Hwang J.M.: ‘Assessment of binocular alignment using the three-dimensional strabismus photo analyzer’, Br. J. Ophthalmol., 2011, 96, (1), pp. 78–82 (doi: 10.1136/bjophthalmol-2011-300305) 21984778 10.1136/bjophthalmol-2011-300305

[8] Toyama T. Kieninger T. Shafait F.: ‘Gaze guided object recognition using a head-mounted eye tracker’. Proc. Symp. Eye Tracking Research and Applications, 2012

[9] Liang Z. Fu H. Chi Z.: ‘Refining a region based attention model using eye tracking data’. Proc. IEEE Int. Conf. Image Processing, 2010

[10] Liu H. Heynderickx I.: ‘Visual attention in objective image quality assessment: based on eye-tracking data’, IEEE Trans. Circuits Syst. Video Technol., 2011, 21, (7), pp. 971–982 (doi: 10.1109/TCSVT.2011.2133770)

[11] Liang Z. Tan F. Chi Z.: ‘Video-based bio-metric identification using eye tracking technique’. Proc. IEEE Int. Conf. Signal Processing Communication Computing, 2012

[12] Kim S. Lombardino L.J. Cowles W.: ‘Investigating graph comprehension in students with dyslexia: an eye tracking study’, Res. Dev. Dis., 2014, 35, pp. 1609–1622 (doi: 10.1016/j.ridd.2014.03.043) 10.1016/j.ridd.2014.03.043 24770469

[13] Orlosky J. Itoh Y. Ranchet M.: ‘Emulation of physician tasks in eye-tracked virtual reality for remote diagnosis of neurodegenerative disease’, IEEE Trans. Vis. Comput. Graph., 2017, 23, (4), pp. 1302–1311 (doi: 10.1109/TVCG.2017.2657018) 28129166 10.1109/TVCG.2017.2657018

[14] Guyton D.L. Moss A. Simons K.: ‘Automated measurement of strabismic deviations using a remote haploscope and an infrared television-based eye tracker’, Trans. Am. Ophthalmol. Soc., 1987, 85, pp. 320–331 3447337 PMC1298782

[15] Schiavi C. Orciuolo M.: ‘Automated measurement of strabismic deviation’, Curr. Opin. Ophthalmol., 1992, 3, (6), pp. 731–734 (doi: 10.1097/00055735-199212000-00002) 10147972 10.1097/00055735-199212000-00002

[16] Pulido R.A.: ‘Ophthalmic diagnostics using eye tracking technology’. Memoire de master non publie, KTH Royal Institute of Technology, 2012

[17] Model D. Eizenman M.: ‘An automated Hirschberg test for infants’, IEEE Trans. Biomed. Eng., 2011, 58, (1), pp. 103–109 (doi: 10.1109/TBME.2010.2085000) 20934943 10.1109/TBME.2010.2085000

[18] Model D.: ‘A calibration free estimation of the point of gaze and objective measurement of ocular alignment in adults and infants’, Doctoral dissertation, 2011

[19] Bakker N.M. Lenseigne B.A.J. Schutte S.: ‘Accurate gaze direction measurements with free head movement for strabismus angle estimation’, IEEE Trans. Biomed. Eng., 2013, 60, (11), pp. 3028–3035 (doi: 10.1109/TBME.2013.2246161) 23399951 10.1109/TBME.2013.2246161

[20] Blaha J. Gupta M.: ‘Diplopia: a virtual reality game designed to help amblyopics’. 2014 IEEE Virtual Reality (VR), 2014, pp. 163–164

